# A *Meloidogyne incognita* effector *MiISE5* suppresses programmed cell death to promote parasitism in host plant

**DOI:** 10.1038/s41598-018-24999-4

**Published:** 2018-05-08

**Authors:** Qianqian Shi, Zhenchuan Mao, Xi Zhang, Xiaoping Zhang, Yunsheng Wang, Jian Ling, Runmao Lin, Denghui Li, Xincong Kang, Wenxian Sun, Bingyan Xie

**Affiliations:** 1 Institute of Vegetables and Flowers, Chinese Academy of Agricultural Sciences, Beijing, 100081 China; 2 Department of Plant Pathology and the Ministry of Agriculture Key Laboratory for Plant Pathology, China Agricultural University, Beijing, 100193 China; 3 College of Life Science, Beijing Normal University, Beijing, 100875 China; 4 Horticulture and Landscape College, Hunan Agricultural University, Changsha, 410128 China

## Abstract

Root-knot nematodes (RKNs) are highly specialized parasites that interact with their host plants using a range of strategies. The esophageal glands are the main places where nematodes synthesize effector proteins, which play central roles in successful invasion. The *Meloidogyne incognita* effector *MiISE5* is exclusively expressed within the subventral esophageal cells and is upregulated during early parasitic stages. In this study, we show that MiISE5 can be secreted to barley cells through infectious hyphae of *Magnaporthe oryzae*. Transgenic *Arabidopsis* plants expressing *MiISE5* became significantly more susceptible to *M. incognita*. Inversely, the tobacco rattle virus (TRV)-mediated silence of *MiISE5* decreased nematode parasitism. Moreover, transient expression of *MiISE5* suppressed cell death caused by *Burkholderia glumae* in *Nicotiana benthamiana*. Based on transcriptome analysis of MiISE5 transgenic sample and the wild-type (WT) sample, we obtained 261 DEGs, and the results of GO and KEGG enrichment analysis indicate that MiISE5 can interfere with various metabolic and signaling pathways, especially the JA signaling pathway, to facilitate nematode parasitism. Results from the present study suggest that MiISE5 plays an important role during the early stages of parasitism and provides evidence to decipher the molecular mechanisms underlying the manipulation of host immune defense responses by *M. incognita*.

## Introduction

Root-knot nematodes (RKNs, *Meloidogyne* spp.) are sedentary endoparasites of more than 3000 species of plants that cause major crop losses on a worldwide scale^1–3^. During parasitism, RKNs establish a close relationship with their host plants. Hatched second-stage juveniles (J2s) penetrate plant roots and then migrate intercellularly toward the root tip^4^. Once they enter the vascular cylinder, and migrate into the differentiating zone of the root, the nematodes puncture several plant cells with their stylet and induce multinucleate giant cells (GCs) and become sedentary^1,5^. The GCs are essential for successful parasitism because this unique feeding structure provides nutrition to the developing worm. Like other obligate parasites, RKNs secrete effector proteins through their stylet into host plant cells to enable successful parasitism. During parasitism, secretions synthesized by esophageal glands play a central role in nematode-plant interactions such as invasion of roots, suppression of host immune defenses, and initiation and maintenance of permanent feeding structures^6–8^.

β-1,4-endoglucanases from the cyst nematode were the first parasitism genes identified that are expressed in esophageal glands; these genes are related to host cell wall degradation^9^. Since then, plant nematologists have tried diverse strategies such as cDNA library screening or developing expressed sequence tags (ESTs) to identify more effectors expressed in esophageal glands with limited success^10,11^. The construction of a cDNA library specific to gland cells that was integrated with Sanger sequencing methods has identified 37 putative *M. incognita* effector proteins^7^, and many of them have been proven to perform different functions in nematode parasitism^12–14^.

A classic view of innate immunity in plant pathogen interactions is described by the “Zig–Zag” model^15,16^. In this model, there are two levels of the immune system that plants have evolved against pathogens and parasites: pathogen-associated molecular patterns (PAMPs) -triggered immunity (PTI), and effector-triggered immunity (ETI). Recent studies have shown that plants detect nematode-associated molecular patterns (NAMP) via pattern recognition receptors (PRRs), such as *Cf-2*, *NILR1* and *PGIP*, causing the activation of PTI responses toward nematodes invasion^17–20^. In turn, to improve pathogenicity, several pieces of evidence indicate that plant-parasitic nematodes (PPNs) deploy various effectors that suppress PTI or ETI during nematode-plant interactions. An esophageal gland secreting effector SPRYSEC-19 from *Globodera rostochiensis* has been shown to interact with the LRR region of SW5-F without eliciting a hypersensitive response (HR), supporting the idea that SPRYSECs could hijack the cellular machinery of the host to modify their target^21^. The *G. rostochiensis* ubiquitin carboxyl extension protein GrUBCEP12, cleaves into free ubiquitin and GrCEP12 *in planta*, and GrCEP12 can suppress Gpa2/RBP-1 mediated programmed cell death (PCD) in *N. benthamiana*^22^. In *Heterodera schachtii*, constitutive expression of 10A06 in *Arabidopsis* lead to the elevated SPDS2 transcript abundance, which increased plant susceptibility to *H. schachtii*^23^. In *Meloidogyne* species, the effector MjTTL5 of *M. javanica* interacts specifically with *Arabidopsis* thioredoxin reductase catalytic subunit (AtFTRc), thereby significantly metabolising plant reactive oxygen species (ROS) and suppressing PTI^24^. Currently, high throughput next-generation sequencing techniques are being applied to elucidate the mechanism of the interaction between nematodes and their host plants. These techniques will lead to a much wider range of PPNs effectors being identified in the coming years.

In this study, we identified a *M. incognita* gene that encodes a zinc-finger protein named MiISE5 and characterized its function in nematode parasitism. MiISE5 can suppress *Burkholderia glumae* induced cell death in *N. benthamiana*, and a comparative transcriptome analysis between the wild type (WT) and the *MiISE5*-overexpressing *Arabidopsis* plants revealed that *MiISE5* may participate in manipulating several pathways during the infection process such as transcriptional regulation, inhibiting expression of multiple marker genes response to various biotic and abiotic stimuli. Furthermore, overexpression of *MiISE5* in *Arabidopsis* also disturbed the host hormone signaling pathways involving jasmonic acid, salicylic acid, auxin and ABA. These lines of evidence suggest that *MiISE5* plays an important role in nematode parasitism.

## Results

### Analysis of *MiISE5* sequence and homologue identification in PPNs

The 1305 bp *MiISE5* (Mi03597) cDNA sequence was isolated by RT-PCR from parasitic J2s of *M. incognita*. This sequence encodes a 435 amino acid sequence, harbored an N-terminal signal peptide of 25 amino acids, and was predicted to contain a nuclear localization signal (NLS) motif from peptide position 36–46, two cys2-his2 zinc finger domain (zf_C2H2) from peptide positions 347 to 372 and 361 to 386 (Fig. S1).

**Figure 1:**
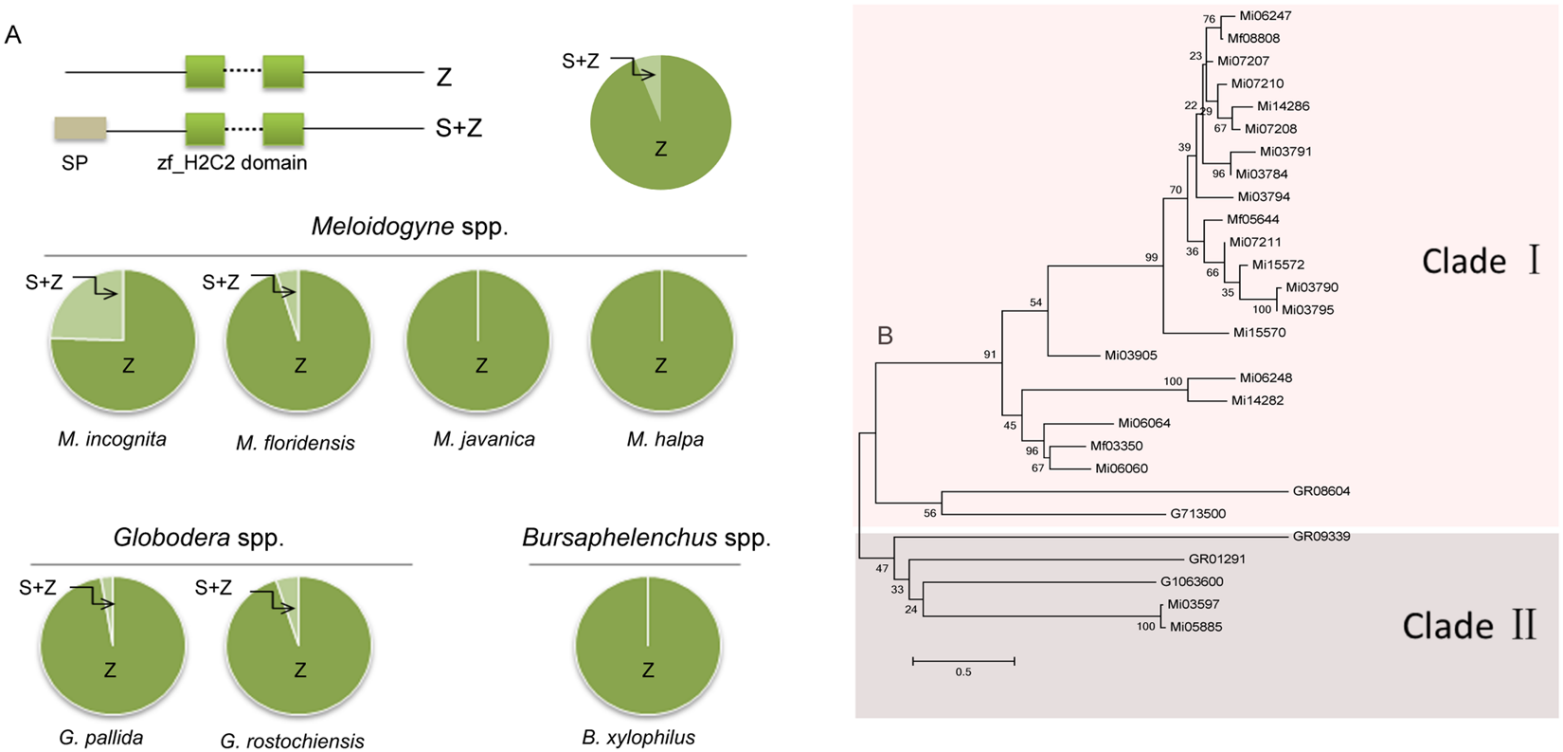
Identification of *MiISE5*-like homologues in PPNs. (**A**) The proportion of secreted zf_C2H2 homologues in seven PPNs. Z: the protein harbors a zf_C2H2 domain. S + Z: the protein harbors a signal peptide (SP) and zf_C2H2 domain, and has no transmembrane domain. (**B**) The phylogenetic tree of secreted zf_C2H2 homologues based on maximum likelihood method. The bootstrap value is given on each node.

Homologues of *MiISE5*-like proteins from seven PPNs species were obtained through a blastp analysis; all 475 homologues contained at least one zf_C2H2 domain, but among them, only 29 proteins contained a signal peptide and had no transmembrane domain (Tables S2, S3). Moreover, those secreted zf_C2H2 homologues were only found in 4 PPNs: *M. incognita*, *M. floridensis*, *G. pallida*, and *G. rostochiensis* (Fig. 1A). A phylogenetic tree was constructed using the maximum likelihood method to examine the relationships among 29 secreted zf_C2H2 homologues. From the phylogenetic tree we can see that the 29 secreted zf_C2H2 homologues are divided into two clades: clade I incorporates most proteins from *Meloidogyne* spp., and clade II incorporates all proteins from *Globodera* spp. and two *M. incognita* proteins (Mi03597 and Mi05885).

### MiISE5 suppresses basal immunity and *B. glumae* induced cell death in *N. benthamiana*

**Figure 2:**
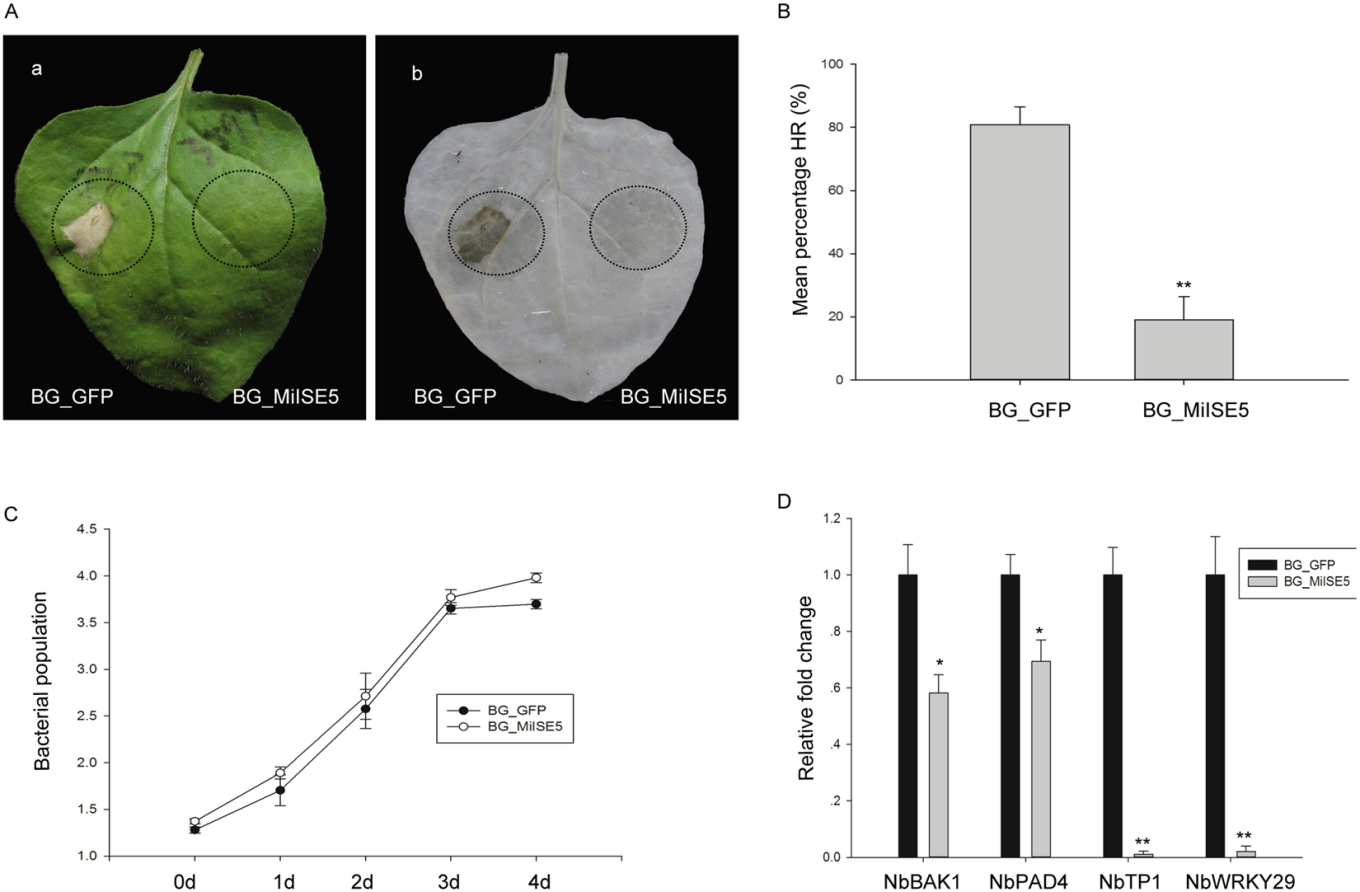
*MiISE5* suppresses *B. glumae* induced cell death and basal defense responses. (**A**) The *N. benthamiana* leaves were photographed within 3 days after *B. glumae* inoculation (a) and then cleared in ethanol (b) to observe the cell death symptoms. The left half leaf sections were injected with *B. glumae* carrying pEDV::GFP and the right half section were injected with *B. glumae* carrying pEDV::MiISE5. (**B**) The average areas of cell death lesions of control GFP and MiISE5. (**C**) Bacterial population [log_10_·(CFU cm^−2^)] in BG_GFP and BG_MiISE5. Bacteria were recovered from inoculated leaves 1–4 days after inoculation. (**D**) Expression of plant defense genes. The expression level of *NbBAK1, NbPAD4, NbTP1, NbWRKY29* were measured by q-PCR in BG_GFP and BG_MiISE5 inoculated leaves at 24 dpi. The actin gene was used as an internal control. Each bar represents the mean ± SD of three independent biological replicates, and the mean values significantly different from the control are demoted by * as determined by an independent samples *t*-test (*P < 0.05, **P < 0.01).

PPNs often impair host immunity through the action of secreted effectors^25,26^. We used a *B. glumae* (BG)-pEDV system to experimentally verify the function of *MiISE5*. *Burkholderia glumae* is a bacterial pathogen with the type III secretion system (T3SS), and it can cause hypersensitive cell death in non-host *N. benthamiana*. The pEDV system utilizes the AvrRps4N (1–137 amino acids of AvrRps4) that is sufficient for T3SS-dependent delivery^27^. First, we cloned a green fluorescent protein (*GFP*) gene into the vector as a negative control, and the HR symptoms were monitored within 3 days after infiltration. Twenty-one inoculated leaves were counted in total. The left half of the *N. benthamiana* leaf that was infiltrated with *GFP* strongly initiated cell death at approximately 3 dpi, and the average area of cell death lesions was 80.9%. However, in the right half of the leaf that was infiltrated with *MilSE5* there was a significant decrease of cell death lesions to 19.1% (Fig. 2A,B). Sometimes, the expression of a gene is lethal for the bacteria, and there was also no HR responses after infiltration. To exclude this possibility, the bacterial growth was monitored for a period of 4 days after inoculation, and the bacterial carrying *MiISE5* can grow as compared to control (Fig. 2C).

Because *BAK1*, *PAD4*, *WRKY29* are working as basal defense responsive markers in *Arabidopsis*^28,29^, we set out to investigate whether MiISE5 suppress the expression of those genes in *N. benthamiana*. The results showed that the expression level of *NbBAK1*, *NbPAD4*, *NbWRKY29* was significantly downregulated in leaf tissues infiltrated by MiISE5 compared to control. Morover, a JA-dependent marker gene *NbTP1*^30^ was also sharply downregulated in the MiISE5 infiltrated leaf zone (Fig. 2D). It means that MiISE5 suppressed basal defense responses in *N. benthamiana*.

### *MiISE5* is expressed specifically in the subventral esophageal gland and is up-regulated in early developmental stages

**Figure 3:**
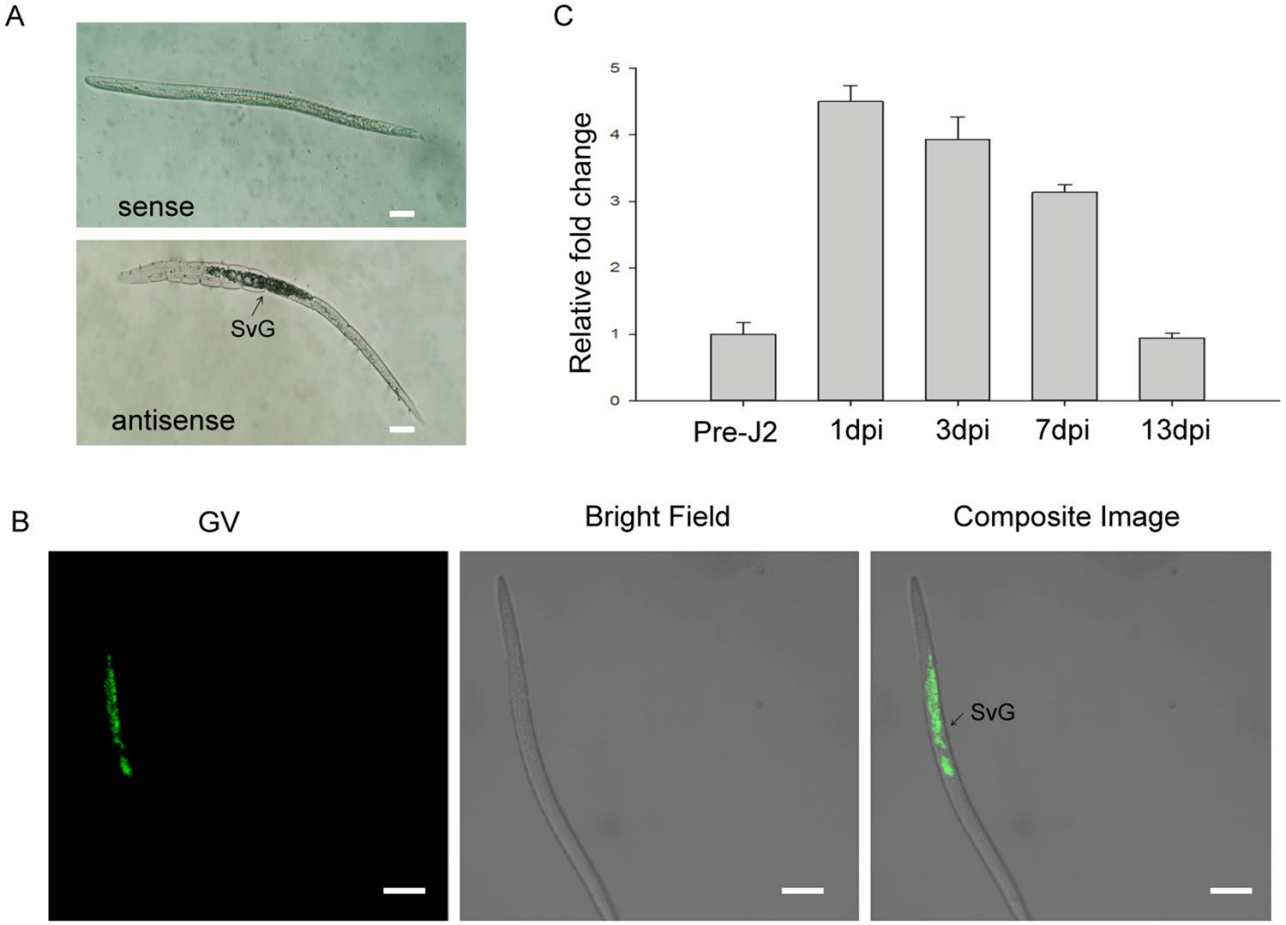
Spatial and developmental expression of *MiISE5*. (**A**) digoxigenin (DIG)-labeled *in situ* mRNA hybridization. (**B**) FITC-labeled *in situ* mRNA hybridization. Both DIG-labeled antisense *MiISE5* cDNA probe and 5′ end labeled with FITC cDNA probe localized *MiISE5* transcripts within the SVGs. Scale bar = 10 μm. (**C**) Developmental expression pattern of *MiISE5*. The relative expression of *MiISE5* was quantified using q-PCR in three different *M. incognita* life stages: pre-J2s, par-J2s (1–3 dpi), and J3s (7–13 dpi). Each bar represents the mean ± SD of three biological replicates; the 18 S gene was used as an internal control. dpi: days postinoculation.

We used *in situ* mRNA hybridization assays to confirm the expressing localization of *MiISE5* in nematode tissues. The hybridization signals were observed in the subventral esophageal gland of the pre-parasitic J2s using the digoxigenin (DIG)-labeled antisense cDNA probes specific for *MiISE5*; no signals were detected when control sense cDNA probes were used (Fig. 3A). Consistent with the result of DIG-labeled *in situ* mRNA hybridization, strong fluorescent signals were observed in the subventral esophageal gland by fluorescence *in situ* hybridization (Fig. 3B).

In addition, we used q-PCR to quantify the expression levels of *MiISE5* during pre-parasitic J2 (pre-J2), parasitic J2 (par-J2), and J3 stages. The sampling time covered the nematode developmental stages of root top invasion, migration in the differentiating vascular cylinder, and the initial formation of permanent feeding sites. The expression levels increased from pre-J2 to par-J2 and then decreased in J3 stages. This developmental expression further supports the idea that *MiISE5* plays an important role during the early stages of plant parasitism (Fig. 3C).

### MiISE5 can be secreted through *M. oryzae* infectious hyphae to plant cells

**Figure 4:**
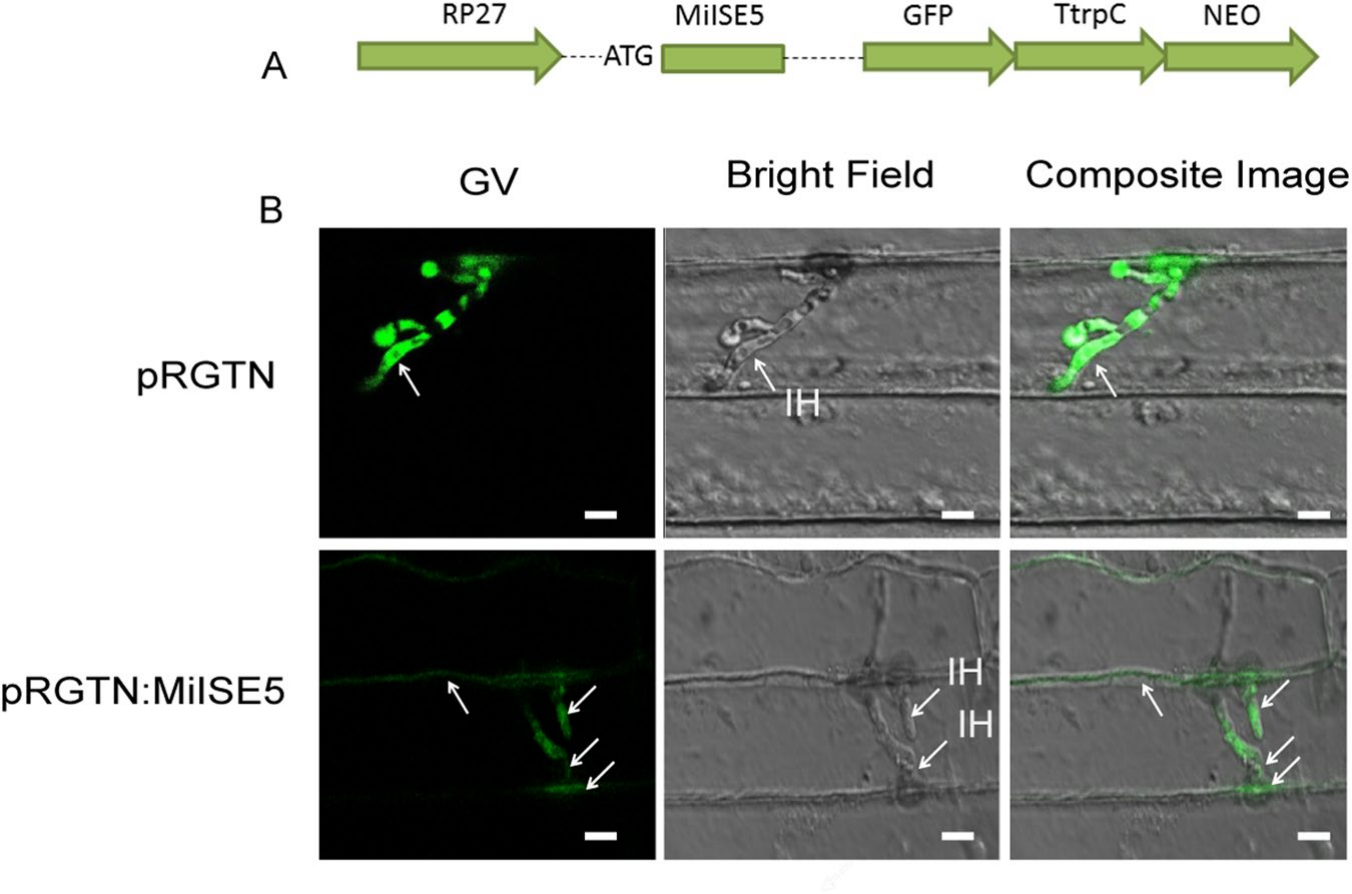
MiISE5 can be secreted through *M. oryzae* infectious hyphae to plant cells. (**A**) Diagrams of the GFP fusion constructs. The MiISE5-GFP fusion construct pRGTN:MiISE5 was under the control of the RP27 promoter. (**B**) GFP signals (white arrows) can be observed in the infectious hyphae and plant cells of the *M. oryzae* transformed with empty vector (pRGTN) and pRGTN:MiISE5, but the GFP signals can only be observed in plant cells penetrated by the infectious hyphae of pRGTN:MiISE5 transformed strain. IH: infectious hyphae. Scale bar = 10 μm.

The *in situ* mRNA hybridization assays showed an subventral esophageal gland expression of *MiISE5*. To further verify that MiISE5 can be secreted into the plant cell, we transformed a *MiISE5-GFP* fusion construct into *M. oryzae* and observed the GFP signals 24 h after inoculating a conidial suspension onto barley leaves. Compared with the *M. oryzae* transformed with empty vector (pRGTN), the GFP signals appeared not only in the infectious hyphae, but also in the plant cells that were penetrated by the infectious hyphae of pRGTN:MiISE5 transformed strains (Fig. 4). These results provided evidence that MiISE5 habor a functional signal peptide, suggested that it could be a potentially secreted protein.

### TRV-mediated *MiISE5* silencing causes diminished nematode parasitism

**Figure 5:**
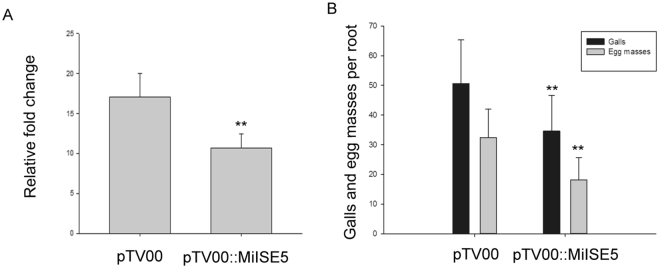
TRV-mediated *MiISE5* silencing effect on nematode parasitism. (**A**) q-PCR assays were used to determine the specificity of RNAi. Each bar represents the mean ± SD of three biological replicates. (**B**) The average number of galls and egg masses per root. Each bar represents the mean ± SD of three biological replicates, and the mean values significantly different from the control are demoted by ** as determined by independent samples *t*-test (P < 0.01).

To assess the role of *MiISE5* in nematode parasitism, we used a TRV-based virus-induced gene silencing system (VIGS) to mediate *M. incognita* gene silencing. To confirm the successful invasion of the virus, we first checked transcripts of the coat protein (*cp*) gene using reverse transcription PCR (Fig. S2) and then randomly selected at least ten plants for q-PCR assays to determine the efficiency of RNAi. The results showed that the transcripts of *MiISE5* were significantly decreased in the par-J2s of plants agroinfiltrated with pTV00:MiISE5 construct compared with the control plants (mean 17.07 vs. 10.71) (Fig. 5A). Importantly, we found that there was a 31.51% reduction of galls and a 44.01% reduction of egg masses in RNAi plants (Fig. 5B).

### *MiISE5* overexpression in *Arabidopsis* promotes susceptibility to nematode parasitism

**Figure 6:**
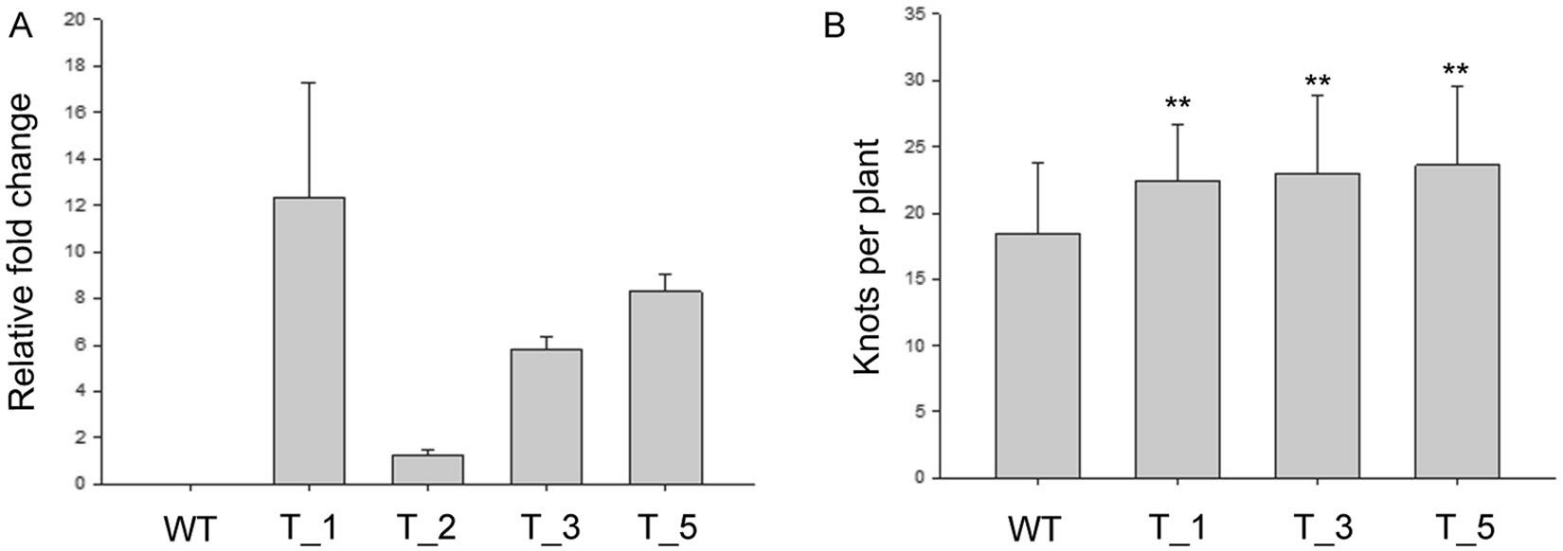
*MiISE5* overexpression in *Arabidopsis* promotes nematode susceptibility. (**A**) q-PCR assays were used to determine the expression of *MiISE5* in four independent transgenic lines. (**B**) The average number of galls per root. Each bar represents the mean ± SD of three biological replicates, and the mean values significantly different from the control are demoted by ** as determined by independent samples *t*-test (P < 0.01).

To determine the role of *MiISE5* on plant phenotype and susceptibility to nematodes, Four independent homozygous *Arabidopsis* lines expressing *MiISE5* were generated. Expression of *MiISE5* in independent transgenic lines was confirmed by q-PCR assays (Fig. 6A); we selected transgenic line T_1, T_3, T_5 for phenotype observation and nematode inoculation. Although there were no obvious changes in morphology, transgenic lines inoculated with *M. incognita* exhibited a 22.11–28.75% increase in the number of galls compared to WT (Fig. 6B). These data indicate that *MiISE5* is an important effector in mediating parasitic success of *M. incognita*.

### MiISE5 was localized in the cytoplasm of plant cell and has no transcriptional activation activity in yeast

**Figure 7:**
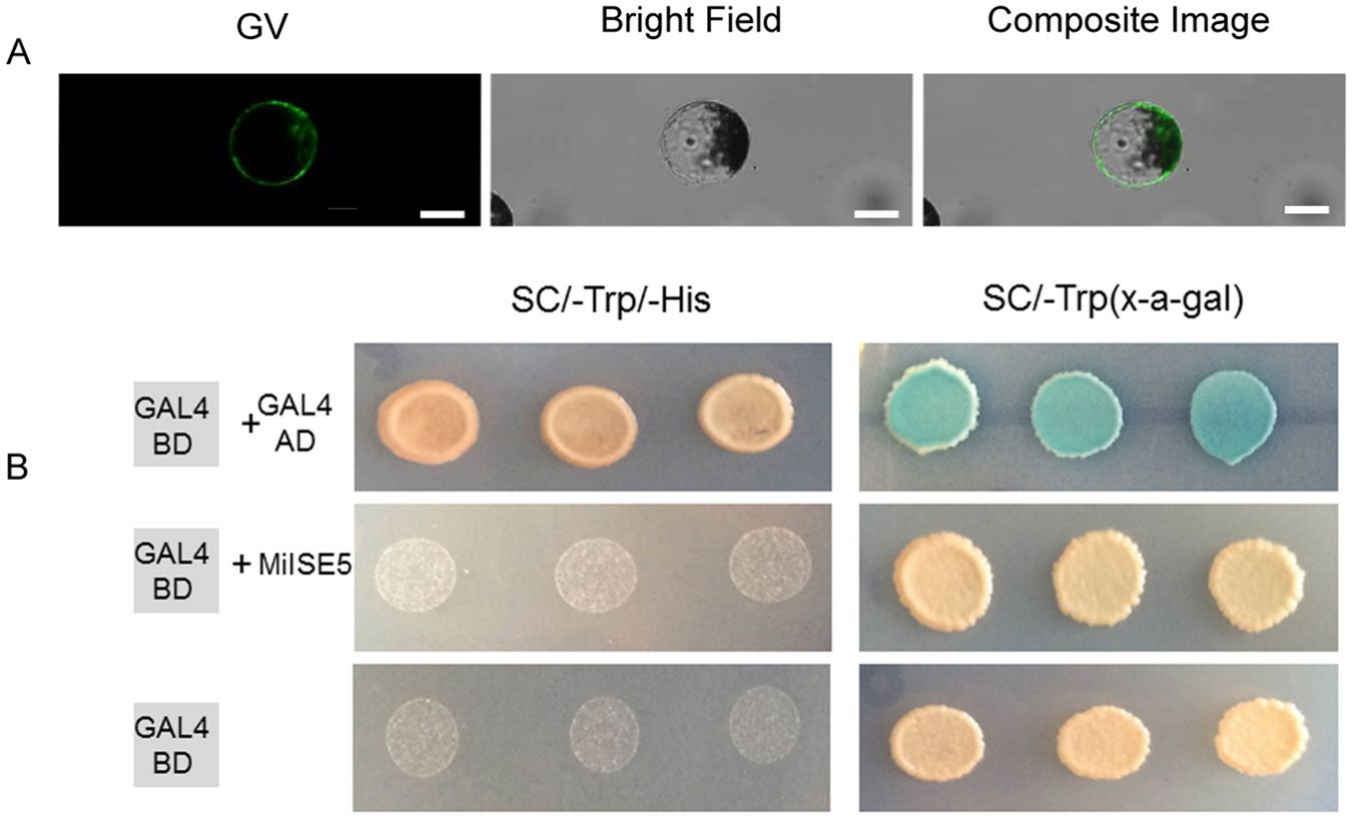
Subcellular localization of transiently expressed MiISE5^Δsp^:GFP in *Arabidopsis* protoplasts and testing of transcriptional activation ability of MiISE5 in yeast. (**A**) MiISE5^Δsp^ was localized to the cytoplasm. (**B**) The MiISE5 was fused with GAL4BD and expressed in yeast cells. GAL4BD and GAL4BD-GAL4AD were used as negative and positive controls, respectively. The blue yeast colonies on synthetic medium without tryptophan (SC/-Trp) with the chromogenic substrate 5-bromo-4-chloro-3-indolyl-αomogalactopyranoside (X-α-Gal) indicate the expression of *MEL-1* gene. The successful growth of yeast colonies on synthetic medium without tryptophan and histidine (SC/-Trp/-His) indicate the expression of the *HIS3* reporter gene. Scale bar = 50 μm.

In order to confirm the subcellular localization of MiISE5, the MiISE5:GFP and MiISE5^Δsp^:GFP was transiently expressed in *Arabidopsis* protoplasts, respectively. Green fluorescence of MiISE5:GFP and MiISE5^Δsp^:GFP was detected in the cytoplasm(Fig. S3, Fig. 7A). We also used a yeast reporter system to test whether MiISE5 could transcriptionally activate gene expression, and the results showed that MiISE5 failed to activate either the expressing of *HIS3* reporter gene or the *MEL-1* gene (Fig. 7B).

### Analysis of differentially expressed genes (DEGs) between *MiISE5* transgenic plants and WT plants

To explore the impact of endogenous expression of *MiISE5* on plant signaling pathways, we performed RNA-seq analysis of WT and *MiISE5*-overexpressing *Arabidopsis* plants. Using the transcript per million fragments(FPKM) method to calculate gene expression levels, we identified 261 genes between the *MiISE5*-overexpressing sample and the WT sample (|log_2_FC| ≥ 1 and FDR ≤ 0.01), of which 227 were upregulated and 34 were downregulated in the *MiISE5*-overexpressing sample (the full list of DEGs is provided in Supplementary Dataset 1).

In order to confirm the results of the RNA-seq analysis, we performed q-PCR assays using independently collected samples that were in the same developmental stage as those used for the RNA-seq analysis. The expression patterns of the 38 randomly selected DEGs were similar to the results from RNA-seq, implying that the transcript value nearly reflected the true expression levels of all expressed genes (Fig. S4, Supplementary Dataset 1).

### Functional annotation analysis of DEGs

**Figure 8:**
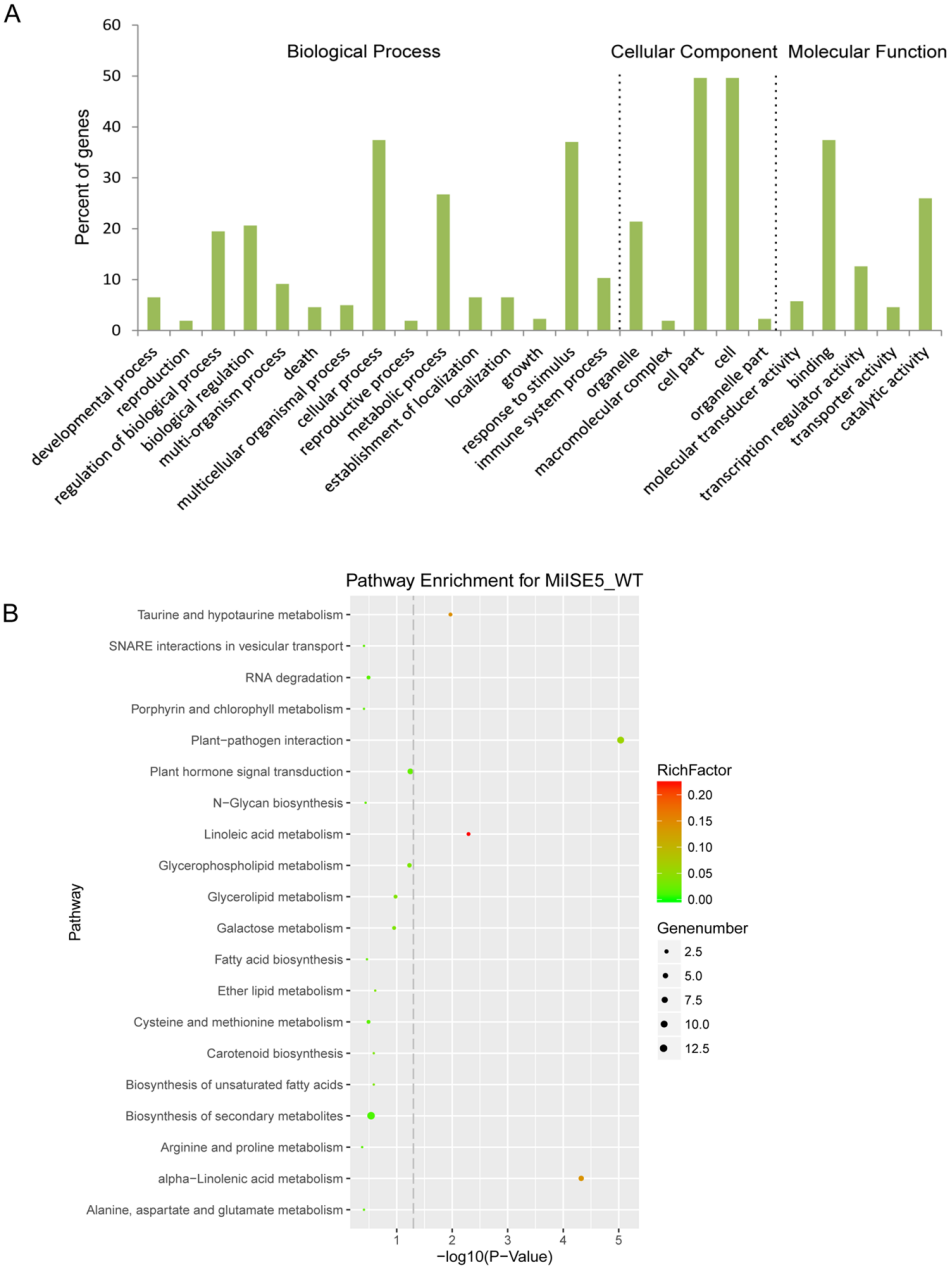
GO and KEGG enrichment of DEGS. (**A**) Gene Ontology (GO) enrichment of DEGs. (**B**) Scatter diagram of KEGG pathways. The y-axis represents the KEGG pathways. The color of the dot represent the “enrichment factor”(RichFactor), which indicates the ratio of the proportion of all genes annotated to a pathway to the proportion of the DEGs annotated to a pathway. The size of the dot indicates the number of DEGs grouped to the pathway. The x-axis represents the *P* value, with points near the left in the figure being more significant.

To further understand the function of these DEGs, gene ontology (GO) term enrichment analysis (FDR ≤ 0.05) was performed. DEGs were categorized into three groups: Biological Process, Cellular Component, or Molecular Function (Fig. 8A). In defense response category (GO:0098542)(Table S5), a number of genes work as negative regulator of plant defense responses, such as transcriptional factor WRKY40, WRKY18 and WRKY48 were upregulated more than eightfold (log_2_FC > 2.5) in the transgenic plants compared to control. Besides, nine genes may paticipate in interfering JA response signaling, such as LOX3 and LOX4, both of them contributed to repressing the biosynthesis of JA. A high expression level of *LOX3* in host contributed to nematode development and reproduction^31^, and the expression of *LOX3* exhibit a twenty eight fold(log_2_FC = 5.31) in the transgenic plants compared to control. Additionally, some JAZ genes were also upregulated in the transgenic plants, and they may play role in suppressing JA responsive signaling.

DEG sequences were mapped to the reference canonical pathways in KEGG. The results of KEGG enrichment analysis indicated that a total of 82 annotated DEGs were assigned to 28 pathways. As shown in Fig. 8B, there were 5 significantly enriched pathways (P ≤ 0.05) of DEGs; the top three pathways were “Plant-pathogen interaction”, “Plant hormone signal transduction” and “alpha-Linolenic acid metabolism” (Table S6). These results implied that MiISE5 play role in plant-nematode interaction and may interfere the hormone signaling pathways to weaken the host immune system.

## Discussion

During nematode-plant interactions, PPNs deliver a variety of effectors into the host tissues using their stylet. These effectors play central roles in facilitating plant parasitism^5^. Previous studies have identified a number of effectors that are relevant to host immunity suppression, such as Mi-CRT, MjTTL5, and MiMsp40 of *Meloidogyne* spp.^24,32,33^; SPRYSEC-19, VAP1 of *G. rostochiensis*^21,22,34^; HgGLAND18 and 30C02 of *Heterodera* spp.^35,36^. According to transcriptomic-based screening, we cloned the full-length *MiISE5* gene and characterized its roles in nematode parasitism, and several lines of evidence indicated that MiISE5 is secreted into host tissues by infective second-stage juveniles. First, *MiISE5* contains a typical N-terminal signal peptide, which could target it to the secretory pathway. Second, *in situ* hybridization indicated that *MiISE5* was expressed in the esophageal gland cells, and MiISE5 can be secreted to the barley cells through *M. oryzae* infectious hyphae. Third, expression of the *MiISE5* gene was intensively upregulated at the early parasitic stage, implying a potential role in parasitism.

Most of the zinc finger proteins were reported to localized in the nucleus of plant cells and work as transcriptional regulators, but it has recently demonstrated that a number of zinc finger proteins can also localized to cytoplasm or plasma membrane, such as OsTZF1, AtTZF2 and AtTZF3. These proteins play important role in celluar functions, including protein-protein interaction, RNA metabolism and ABA/JA responses^37–40^. The subcellular localization verified that MiISE5 was expressed in cytoplasm, and no transcriptional activation was observed in yeast system. Both results indicated that MiISE5 was probably not work as a transcriptional regulator, and we speculated that it may play roles in protein-protein interaction or post-translational modification in host cells.

RNAi is a powerful tool that we used to knock out the expression of candidate effector genes and to examine the subsequent effect on nematode development and nematode establishment in its host^41^. In this study, we produced TRV-mediated gene silencing pepper plants to evaluate the effect of *MiISE5* on nematode parasitism. Compared to the control plants, there was a significant reduction in the number of knots and eggs in RNAi plants. Additionally, we also generated transgenic *Arabidopsis* overexpressing *MiISE5*. Consistent with an important role in infection, the overexpression of *MiISE5* increased *M. incognita* pathogenicity: the transgenic line showed an increased number of females compared with the WT lines. Together, these results provide direct evidence that *MiISE5* is involved in plant parasitism.

PPNs have a variety of interactions with plants when exposed to a variety of host defenses^42^. PTI is the first immune response against PPNs invasion; successful PPNs can secrete effectors that with interfere PTI, often resulting in a fast, localized plant cell death (PCD) called the HR. The initiation of cell death can restrict the expansion of the nematode-induced feeding structure, and there is evidence that some nematode effectors may suppress these HR responses^15,16^. The *M. graminicola* effector MgGPP, which accumulates in host giant cell nuclei during parasitism, suppresses plant immunity responses by *in planta* glycosylation. The N-glycosylation of MgGPP is in concert with plant proteolysis. Moreover, the expression of MgGPP in *N. benthamiana* can suppress cell death induced by Gpa2/RBP-1^25^. To validate the effect of *MiISE5* in suppressing plant immunity, we experimentally demonstrated that *MiISE5* consistently attenuated *B. glumae*-triggered HR and basal defense in *N. benthamiana*, indicating that *MiISE5* can suppress cell death by modulating the signaling pathway of PTI and ETI. This phenomenon is similar to MgGPP and some other effectors such as PITG_18215 and *Msp40* in previous studies^33,43^.

Plant immunity depends on a crosstalk network of the hormone signaling pathway^44^. The jasmonic acid (JA) and salicylic acid (SA) as important signaling molecules in plant defense responses towards parasites, and rencent studies indicate that JA play a crucial role during plant-pathogen interactions in roots^18,45,46^. Integrated the GO and KEGG enrichment analysis, we speculated that *MiISE5* may mainly act in repressing JA-dependent defenses to facilitate parasitism. The up-regulation of *CYP94B3*, *CYP94C1*, *LOX3*, and *LOX4* suppress the accumulation of JA/JA-Ile, and the decreased level of JA/ JA-Ile may stop JAZ proteins from being targeted by SCF^COI1^ E3 ubiquitin ligase to 26 S proteasome^47^. Upregulated expression of JAZ genes led to repression of MYC genes, which then repressed JA responsive genes. Our results are in agreement with those obtained by Nahar *et al*. and Fujimoto *et al*.^45,46^. However, *Arabidopsis* transcription factors *WRKY18*, *WRKY40*, *WRKY33*, and *WRKY48* also had high expression levels in the transgenic plants, and these genes may act in a feedback repression system controlling basal defense^48–50^. Furthermore, the expression of negative regulators of nematodes or other pathogens, such as *AP2C1*, *PCC1*, and *ERF104*^51–53^ were also upregulated in the transgenic plants (Fig. S5).

In the present study, we obtained a *M. incognita* effector *MiISE5* and predicted its secreted homologues in other PPNs. We speculated that once MiISE5 is secreted from the stylet to the host tissues, it plays an important role during the early stages of parasitism by modulating multiple signaling pathways to enhance nematode parasitism and suppress the resistance responses of the host plant. Although the host target of MiISE5 is not yet clear, our studies provide important evidences to explore the mechanism of plant-nematode interaction.

## Materials and Methods

### Nematode and plant materials

*Meloidogyne incognita* nematodes were propagated on 3-week-old pepper (*Capsicum annuum*, Qiemen) plants in a greenhouse. The handpicked eggs were hatching with water in a culture dish at 26 °C, and the infective J2s were collected for infection assays or RNA extraction 1 or 2 days later. The *N. benthamiana* and barley were cultivated in a growth chamber under controlled condition (16 h light/8 h dark) at 25 °C, and the *A. thaliana* plants of the Col-0 ecotype were cultivated under controlled condition (16 h light/8 h dark) at 23 °C.

### Sequence analyses

Nematodes genome and transcripts of PPNs used in the homologue analysis of this study except *M. javanica* were download from NCBI (Table S1). The predictions of the signal peptide were performed using SignalP 4.1 (http://www.cbs.dtu.dk/services/SignalP/), predisi (http://www.predisi.de/) and phobius (http://phobius.binf.ku.dk/). The prediction of transmembrane and conserved domain were performed using TMHMM (http://www.cbs.dtu.dk/services/TMHMM-2.0/) and NCBI CD-Search (https://www.ncbi.nlm.nih.gov/Structure/cdd/wrpsb.cgi). A phylogenetic analysis of 29 secreted homologues was performed using the maximum likelihood method with MEGA 5 software.

### Fluorescence *in situ* hybridization

A 25 bp specific cDNA region (CCTCAGACATCTGTTGGGGCATTTC) was selected for hybridization probe, whose 5′ end labeled with fluorescein isothiocyanate (FITC, Sangon Biotech). The protocol of this study referred to the method of fluorescence *in situ* hybridization used in visualization of bacteria with appropriate modification^54,55^. Before the hybridization, we performed series of pretreatments to ensure that the probe can easily go inside of nematodes. The collected J2s nematodes were firstly disinfected with 0.1% (wt/vol) benzalkonium chlorides for 1 min, and washed with 0.85% (wt/vol) NaCl twice for 2 min. Then the nematodes were fixed in glacial acetic acid containing 50% (vol/vol) ethanol for 10 min, and dehydrated them in a graded ethanol series (50, 75, and 98% ethanol [vol/vol], 2 min). Subsequently, the nematodes were transferred in buffer consisting of a 1:1 mixture of methanol and PBT (8.70 g of NaCl, 1.63 g of Na_3_PO_4_, 1 ml of Tween-20 per liter) for 10 min, then re-suspended them in PBT with 1% (vol/vol) methanol for 5 min and washed them with PBT twice for 2 min.

After pretreatment, the nematodes were suspended in phosphate-buffered saline (PBS) containing 0.3% (vol/vol) triton X-100 and then treated with hybridization buffer which consisted of 1 M Tris-HCl (PH = 8.0), 5 M NaCl, 20% (wt/vol) SDS, 30% (vol/vol) formamide for 10 min, and then 10 μM FITC-labelled probe was applied to nematodes for 12 h and then rinsing twice with PBS containing 0.3% (vol/vol) triton X-100. 10 μl of the nematodes suspension was mixed to 10 μl mouting fluid on a glass slide (90 ml of glycerine, 10 ml of PBS, 1.25 g of DABCO), and observed under a confocal microscope (Zeiss LSM700, Germany).

### Digoxigenin-labeled *in situ* hybridization

Digoxigenin-labeled sense and antisense cDNA probes were synthesized using the primer DIG_MiISE5_F and DIG_MiISE5_R. The primers used in this study were listed in Table S4. Pre-parasitic stage J2s of *M. incognita* were processed for *in situ* hybridization as previously described^6,11^. Hybridization signals within the nematode were detected under a light microscope (Olympus, Germany).

### Validation of the secretion of MiISE5 using *M. oryzae* transformation and infection assays

The vector pRGTN was used for protoplasts transformation to validate the secretion of MiISE5. The PCR products of coding sequence of *MiISE5* were digested with Hind III and BamH I and inserted into pRGTN digested with the same enzymes for generating pRGTN:MiISE5. The mycelia of *M. oryzae* strain P131 collected from 2-day-old cultures in complete medium (CM) were used for the isolation of protoplasts, and the protoplasts were transformed using PEG method described by Park G *et al*. and Chen X L *et al*.^56,57^.

The conidia of the transformed *M. oryzae* were harvested from 10-day-old cultures and then resuspended in 20% (vol/vol) tween-20 to a concentration of 5 × 10^5^ conidia/ml. conidial suspension were dotted onto barley leaves and then incubated in a dark chamber keeping moist at 28 °C. 24 h after inoculation, the barley leaves were observed under a confocal microscope (Zeiss LSM700, Germany).

### Quantitative RT-PCR (q-PCR) analysis of *M. incognita* and Arabidopsis genes

Total RNA was extracted from 50 mg *M. incognita* using RNAprep pure Tissue Kit (Tiangen, China) or 100 mg frozen plant tissue using RNAprep pure Plant Kit (Tiangen, China). The cDNA was synthesized using FastQuant RT Kit with gDNase (Tiangen, China). Quantitative RT-PCR was performed using the primer pairs qMiISE5_F2/qMiISE5_R2 and 18 S_F/18S_R for *MiISE5* and the internal control gene 18S (Genbank accession no. U81578). Each 25 μl reaction mixture was prepared using SYBR Premix Ex Taq^TM^II(TaKaRa, Japan) on a BIO-RAD CFX96 (BIO-RAD, USA). q-PCR program was as 95 °C for 5 min and 40 cycles of 95 °C for 30 s and 60 °C for 30 s. Gene expression changes were calculated using the 2^−ΔΔCT^ method^58^. At least three independent experiments were performed, with four technical replicates for each reaction.

### TRV mediated silencing of *MiISE5*

A 283 bp fragment of the *MiISE5* gene was amplified by PCR using the primer pairs TRV_MiISE5b_F and TRV_MiISE5b_R. Using In-Fusion® HD Cloning Kit (Clontech, USA), the PCR products were cloned into a pTV00 digested with *BamH* I and *Hind* III for generating pTV00::MiISE5. The constructs were then transformed into the *Agrobacterium tumefaciens* GV3101 using electroporation (Bio_Rad Gene Pulser Xcell®, USA). The pepper plants were inoculated by GV3101 carrying the corresponding constructs using procedures as previously described^59,60^, the infiltrated pepper plants were inoculated with 800 *M. incognita* J2s per plant. The pepper roots were washed and the number of galls and egg masses were counted 6 weeks after inoculation. At least 30 pepper plants were used for each treatment, and three independent experiments were performed.

The primer pairs TRV_CP_F and TRV_CP_R were used to verify the successful invasion of the *Tobcco rattle virus* (TRV), and we collected 200 par-J2s at 3 dpi from the plants which were successfully infected by virus for RNA extraction. q-PCR was performed to measure the silencing efficiency of *MiISE5* using the primer pairs qMiISE5_F2/qMiISE5_R2 and 18S_F/18S_R for *MiISE5* and the internal control gene, and three independent experiments were performed. Statistically significant differences between treatments were determined by independent samples *t*-test (P < 0.05) with SPSS.

### Transgenic Arabidopsis plants generation and nematode infection assays

For *Arabidopsis* plants overexpression, the coding sequence of *MiISE5* gene minus signal peptide was cloned into the vector pEGAD harboring the BASTA selectable marker. The generated construct pEGAD::MiISE5 was then introduced into *A. tumefaciens* GV3101 and used to transform *Arabidopsis* Col-0 ecotype^61^. Transformed T1 seeds were sown on soil and were screened by spraying the herbicide BASTA two weeks later to select for transgenic plants. The spraying was repeated thrice. Homozygous T3 seeds were collected from T2 lines after a segregation analysis and were used in this research. 4 weeks old *Arabidopsis* were inoculated using 400 J2 of *M. incognita* per plant, and the wild type Arabidopsis(Col-0) was used as a negative control. 42 days after inoculation, the number of RKN females was counted for at least 30 plants of each line. Three independent experiments were perfomed. Statistically significant differences between treatments were determined by independent samples *t*-test (P < 0.05) with SPSS.

### Cell death suppression and measurement of bacteria growth rate

The suppression of cell death elicited by *B. glumae* on *N. benthamiana* leaves was performed according to Shailendra Sharma’s method^27^. The *MiISE5* and *GFP* coding sequences were cloned into the vector pEDV5^62^. The confirmed constructs were then introduced into the *B. glumae* by electroporation. *Nicotiana benthamiana* plants were infiltrated with bacterial inocula (suspended in a 0.9% NaCl) of OD_600_ = 0.4 using a 1 ml needleless syringe. The left half of leaf was injected with *B. glumae* carrying pEDV::GFP, and the right half was injected with *B. glumae* carrying pEDV::MiISE5, three leaves per plant and 4 plants were used for each biological replicate, and at least three independent experiments were performed. Plants were kept at 25 °C. Cell death was observed within 3 days after infiltration, and leaves were then cleared in ethanol to visualize the HR. For bacteria population counting, Three 1.0 cm^2^ leaf discs from different bacteria infiltrated plants were collected as one sample, after sterilizing in 75% ethanol, the leaf discs were gound in 0.9% NaCl and plated gradient dilution on LB agar plates containing 25 mg/L Gentamicin to determin the CFU cm^−2^. The plates were incubated at 28 °C for three days until colonies could be counted. Three independent experiments were performed.

### Subcelluar localization

The coding sequence (without stop codon) of MiISE5 and MiISE5^Δsp^ was cloned into pCAMBIA1302 to generate the MiISE5:GFP and MiISE5^Δsp^:GFP, respectively. The preparation of *Arabidopsis* protoplasts and PEG mediated transfection were performed as previously described^63^.

### Yeast transcriptional activation assay

To determine the transcriptional activity, we used a system based on the Matchmaker Gold Yeast Two-Hybrid Kit (Clontech, Japan). the coding sequence of *MiISE5* gene minus signal peptide was cloned into pGBKT7. Constructs were introduced into *Saccharomyces cerevisiae* strain AH109 using the Frozen-EZ Yeast Transformation II kit (Zymo Research, USA). After transformation, yeast was plated on synthetic medium without tryptphan (SC/-Trp). Three independent yeast colonies were suspended in sterile water and dotted on SC/-Trp with X-α-Gal and SC/-Trp/-His, respectively, to observe the activation of the *HIS3* and *MEL-1* reporter genes. All experiment were replicated three times and three independent transformations were used for each replicate.

### Transcriptome analysis

The 14 days *MiISE5* overexpressing *Arabidopsis* plants grown on Murashige and Skoog (MS) solidified medium containing 2% sucrose were used for cDNA libraries construction. The WT plants were used as negative control. Illumina technology generating 280 bp read pairs was employed to sequence the cDNA libraries. Low-quality data that had a qual value of less than 20 and consisted of short reads (length < 35 bp) were filtered from the raw data. Clean reads were aligned to the *Arabidopsis* genome using bowtie2 and tophat^64^, htseq-count was then used to calculate the expected fragments per kilobase of FPKM mapped, and the edgeR package in R is applied to identify DEGs with fold-change ≥2.0 or ≤−2.0 (|log2FC| ≥ 1) and Benjamini-Hochberg corrected false discovery rate (FDR) ≤ 0.01. We used AgriGO (http://bioinfo.cau.edu.cn/agriGO/) and KOBAS 3.0 (http://kobas.cbi.pku.edu.cn) to perform GO functional enrichment analysis and KEGG enrichment analysis with default parameters.

## Electronic supplementary material

Supplementary information

Supplimentary dataset 1
