# Supplementary material for: A Meloidogyne incognita effector MiISE5 suppresses programmed cell death to promote parasitism in host plant
Source: Sci Rep. 2018 May 8;8:7256. doi: 10.1038/s41598-018-24999-4 (PMC5940819; doi:10.1038/s41598-018-24999-4)
Supplement: Supplementary file 1 — Supplementary information [file 41598_2018_24999_MOESM1_ESM.pdf]

Supplementary information

A *Meloidogyne incognita* effector *MiISE5* suppresses programmed cell death to promote parasitism in host plant

Qianqian Shi[a,b,#], Zhenchuan Mao[a,#], Xi Zhang[a,c#] , Xiaoping Zhang[a], Yunsheng Wang[a], Jian Ling[a], Runmao Lin[a,c], Denghui Li[a], Xincong Kang[d], Wenxian Sun[b], Bingyan Xie[a,*]

[a]*Institute of Vegetables and Flowers, Chinese Academy of Agricultural Sciences, Beijing 100081, China*

[b]*Department of Plant Pathology and the Ministry of Agriculture Key Laboratory for Plant Pathology, China Agricultural University, Beijing 100193, China*

[c]*College of Life Science, Beijing Normal University, Beijing 100875, China*

[d]*Horticulture and Landscape College, Hunan Agricultural University, Changsha 410128, China*

Authors: #Qianqian Shi, #Zhenchuan Mao, #Xi Zhang are equal contributors;

Corresponding author:

**E-mail addresse:* xiebingyan@caas.cn (Bingyan Xie).

Supplementary Table S1. The platforms about nematodes genome and transcripts of RKNs used in the homologue analysis of this study.

Species	Blast server links
Meloidogyne incognta	https://www.ncbi.nlm.nih.gov/genome/?term=meloidogyne+incognita
Meloidogyne hapla	https://www.ncbi.nlm.nih.gov/genome/?term=meloidogyne+hapla
Meloidogyne floridensis	https://www.ncbi.nlm.nih.gov/genome/?term=meloidogyne+floridensis
Meloidogyne javanica	From the transcriptional sequencing database of our lab
Globodera pallida	https://www.ncbi.nlm.nih.gov/genome/?term=Globodera+pallida
Globodera rostochiensis	
Bursaphelenchus xylophilus	https://www.ncbi.nlm.nih.gov/genome/?term=Bursaphelenchus+xylophilus

Supplementary Table S2. The number of homologues contain only zf_C2H2 domain and homologues which contain both SP, zf_C2H2 domain and have no transmemberance domain.

species	No of homologues contain zf_C2H2 domain	No of homologues contain SP, zf_C2H2 domain, and have no transmemberance domain
Meloidogyne incognta	86	21
Meloidogyne hapla	41	0
Meloidogyne floridensis	77	4
Meloidogyne javanica	82	0
Globodera pallida	74	2
Globodera rostochiensis	56	3
Bursaphelenchus xylophilus	59	0

Supplementary Table S3. The corresponding Gene ID and gene information of the secreted homologs of MiISE5 in the Bayesian phylogenetic tree.

Gene name	Corresponding gene ID	No of zf_C2H2 domain	Amino acid sequence length	Organism
Mi07211	Minc07211	2	406	*Meloidogyne incognta*
Mi14286	Minc14286	2	421	*Meloidogyne incognta*
Mi07210	Minc07210	4	422	*Meloidogyne incognta*
Mi03794	Minc03794	2	400	*Meloidogyne incognta*
Mi15572	Minc15572	2	345	*Meloidogyne incognta*
Mi06247	Minc06247	2	368	*Meloidogyne incognta*
Mi07208	Minc07208	2	435	*Meloidogyne incognta*
Mi07207	Minc07207	2	481	*Meloidogyne incognta*
Mi03790	Minc03790	2	503	*Meloidogyne incognta*
Mi03795	Minc03795	2	245	*Meloidogyne incognta*
Mi15570	Minc15570	1	387	*Meloidogyne incognta*
Mi03791	Minc03791	2	222	*Meloidogyne incognta*
Mi03784	Minc03784	2	227	*Meloidogyne incognta*
Mi06248	Minc06248	1	404	*Meloidogyne incognta*
Mi14282	Minc14282	1	211	*Meloidogyne incognta*
Mi03905	Minc03905	2	242	*Meloidogyne incognta*
Mi06064	Minc06064	1	180	*Meloidogyne incognta*
Mi06060	Minc06060	1	326	*Meloidogyne incognta*
Mi03597	Minc03597	2	434	*Meloidogyne incognta*
Mi05885	Minc05885	2	430	*Meloidogyne incognta*

Mf05644	maker-nMf.1.1.scaf05644<br>-augustus-gene-0.6-mRNA-1	2	315	*Meloidogyne floridensis*
Mf03350	maker-nMf.1.1.scaf03350<br>-augustus-gene-0.5-mRNA-1	1	324	*Meloidogyne floridensis*
Mf01483	genemark-nMf.1.1.scaf01483<br>-processed-gene-0.6-mRNA-1	2	1044	*Meloidogyne floridensis*
Mf08808	maker-nMf.1.1.scaf08808<br>-snap-gene-0.5-mRNA-1	2	276	*Meloidogyne floridensis*
GR01291	GROS_g01291.t1	2	344	*Globodera rostochiensis*
GR08604	GROS_g08604.t1	2	423	*Globodera rostochiensis*
GR09339	GROS_g09339.t1	1	827	*Globodera rostochiensis*
G713500	GPLIN_000713500	2	161	*Globodera pallida*
G1063600	GPLIN_001063600	1	929	*Globodera pallida*

Supplementary Table S4. Primers used in this study (The lowercase regions represent the fusion sequence, the underlined regions represent cleavages sites of restriction enzymes).

Primers	Sequence	Usage
DIG_MiISE5_F	ATGCTTTGTGGTCCAAGTG	*In situ* hybridization
DIG_MiISE5_R	TTGTACTTTTTCTTTATGAATCTTC	
TRV_MiISE5b _F	tagaactagtggatccTTTATTTGTCGTTATTGCGGAC	RNA silence
TRV_MiISE5b_R	cggtatcgataagcttATTTACTAGCATTTTTGCGTGG	
TRV_CP_F	ACTCACGGGCTAACAGTGCT	Virus invasion detection
TRV_CP_R	GACGTATCGGACCTCCACTC	
pEGAD_MiISE5_F	CCGGAATTCATGCTTTGTGGTCCAAGTG	*Arabidopsis* overexpression
pEGAD_MiISE5_F	CGCGGATCCTTGTACTTTTTCTTTATGAATCTTC	
pEDV_MiISE5_F	ACGCGTCGACATGCTTTGTGGTCCAAGTG	Suppression of HR
pEDV_MiISE5_R	CGCGGATCC TTGTACTTTTTCTTTATGAATCTTC	
pEDV_GFP_F	ACGCGTCGACATGGTGAGCAAGGGCGAG	
pEDV_GFP_R	CGCGGATCCCTTGTACAGCTCGTCCATGCC	
pRGTN_MiISE5_F	CCCAAGCTTATGACTATTATGAATGTTTTGCTC	Validation of the functionality of signal peptide
pRGTN_MiISE5_F	CGCGGATCCTCTTCAATTTACTAGCATTTTTG	
pCAMBIA_MiISE5_F	CATGCCATGGATGCTTTGTGGTCCAAGTG	
pCAMBIA_MiISE5_R	CTAGACTAGTTTGTACTTTTTCTTTATGAATCTTC	Subcellular localization
pCAMBIA_MiISE5_F	CATGCCATGGATGACTATTATGAATGTTTTGCTC	
pCAMBIA_MiISE5_R	CTAGACTAGTTCTTCAATTTACTAGCATTTTTG	
pGPK_MiISE5_F	CATGCCATGGATGCTTTGTGGTCCAAGTG	Yeast transcriptional activation assay
pGPK_MiISE5_R	CGCGGATCCTTGTACTTTTTCTTTATGAATCTTC	
qMiISE5_F2	ATCGCCTCGCTTTCATTCTCC	q-PCR
qMiISE5_R2	TTGTAGTCTGGTTGGCTGGTG	

NbBAK1-F	TACTTCGTTTATTGGGCTTTTGC
NbBAK1-R	GATCTGATTCTGGTCGCTCTCTT
NbPAD4-F	TGAGCTGTGCCATTCTCAAC
NbPAD4-R	TCCTCGTAGAGCCACTCGAT
NbWRKY29-F	TGGCTTCTTCTATTTCTTTTCCTA
NbWRKY29-R	ACTCTCTTCTGCTCATTCTTCCTT
NbTP1-F	ACTTTCGAATGCGATCCAAG[30]
NbTP1-R	TCAACCACTTTGCTGCCATA[30]
AT1G17420-F	TGCTCAACACGCCGCTCT
AT1G17420-R	CCGATCTCTGCCGCAAATC
AT3G45970-F	TGTGCTTATGGCTCTATGGCTAC
AT3G45970-R	AAGGACAAGATCGGTTTGGTTAC
AT2G34600-F	CGGTAACGGTGGTAAGGGG
AT2G34600-R	CGGTAACGGTGGTAAGGGG
AT1G17380-F	TTCCCTCCATCGATTCTTTGC
AT1G17380-R	CGGTCTTTGTGACGACTGTCCT
AT5G13220-F	CGTTCGGTTCCGTCTACTCC
AT5G13220-R	GCTGCTTCATTAGCGACCTTC
AT1G72520-F	ATTTTGGCTGAGGACTTCGCT
AT1G72520-R	ACTCGTTCTATGTTGACCGGG
AT3G48520-F	CTGAACCGGGGAGTACACGAC
AT3G48520-R	ACCTTAAGCCCACCAGCCATA
AT2G27690-F	GTCGGGATACGGTAGCTGCTG
AT2G27690-R	GCGAATTTGGAATCGAATTGA
AT5G45340-F	CTCCGGTTTGTTTCTCACTCTCT
AT5G45340-R	TCCGTATCTTCTCTGTTTTGCTG

AT1G80840-F	GTTATTGGCGGAGTGTCGGAG
AT1G80840-R	GGAGCACAAGCACATTTGAAGT
AT5G49520-F	TTCATTCGATGCCTTTCCTCTC
AT5G49520-R	TGGCTTAGTACCCTTTTGCTCTT
AT4G31800-F	GGAGGTCTCAGTTTTGGCTTCT
AT4G31800-R	CGGTATATCGGTCTGCTCGATT
AT4G23810-F	ACAAGACACCAGAGTCAAACCAGC
AT4G23810-R	ACAACGTCGAAGATCGGAGAAC
AT5G62520-F	AGAGGCGGTGCAGATGAAAC
AT5G62520-R	AGATACAATCCACGGCCAAAAG
AT2G22500-F	AGGGTTTTGCTGAAGGAGGAATAG
AT2G22500-R	ACAGAGTTTGACGAAGAACGGTG
AT4G17490-F	GGAAGTGACGGTGGTTGAGAA
AT4G17490-R	CAAACGGTGGGTGTGGAGATA
AT5G03720-F	ACTTCAAGCGATCCAAAAGGC
AT5G03720-R	CCCAGCAGACATCAGGCAAAC
AT1G76650-F	CCGGAGAGATACAACAACATAACA
AT1G76650-R	GAGAAAATTCCTCAAAATCCAACA
AT1G73805-F	GCGAAAGTTGCGAAAGGGAG
AT1G73805-R	TTGATGTGGCGAGAGGAGAGC
AT1G66400-F	TAGGAGCTCTCTCTCCAAACGC
AT1G66400-R	CAACCATCACCATCAGAATCAA
AT2G41010-F	ATGGTGACTTCGGAGGGATTAG
AT2G41010-R	GGAGTGAGAGTATGAGGGGCTG
AT4G08040-F	TCATGGGGAAAATCAGAGAGAAC
AT4G08040-R	CAAACCCTGGATAATAAGGTGCA

AT3G50930-F	CTTCACTCTCTCCCCAGAAAACA
AT3G50930-R	TGGACCGTACAGCAAGTAACCTC
AT5G03350-F	CTTTGGTCCACTCCAGTCCCT
AT5G03350-R	TGTTGGTTTTGTTGAATATCCCG
AT5G26920-F	TTACAATGCAAACACTCCGGG
AT5G26920-R	TGAAAGTCGATGTGCTGCGAT
AT3G59080-F	AAATATGATGTTCGGTTGCGGT
AT3G59080-R	GAAGTGAAGTTCAGATTCGGGTG
AT3G23250-F	AAAGAACCCACCAAGGAACGG
AT3G23250-R	TACCAGACTCAAGTGTTGCCACC
AT3G50060-F	TCGTCTCCTGTTGCTCAGCTG
AT3G50060-R	CGCGTTGTTGTTATTGTTATGGC
AT5G62470-F	AAGCACTAACAAAGGTCAATGGG
AT5G62470-R	TGGTGTCGGTAATGATGAAGATG
AT2G30020-F	AACCTCAGACCTCTTCTCCTTCC
AT2G30020-R	ACCATCACCTTCTCTCTCAACCT
AT4G24380-F	AAACTCTCTCCCTCCACTTTTTAGG
AT4G24380-R	TTCACCATTCTTGTCTTCCTCCAT
AT3G22231-F	GGCCGTACACAAGTCCGC
AT3G22231-R	GCCGCAGCAGAAGATACACTC
AT5G61600-F	AGGCCCTAGAACCATCACCAA
AT5G61600-R	CCAAGCCAGATCCTACAACCC
AT1G20823-F	CTTGGTTGTTGCCAGGTGTCA
AT1G20823-R	CATCTTCGCCTTGCTTGATTC
AT2G35930-F	GAACCACGGAGCAGCCATAG
AT2G35930-R	ACCTTCCCACCGACAACAAAA

AT4G03510-F	TCTGTGGTCACCTCTTTTGCTG	
AT4G03510-R	AACCGGTCCTACGGGTCTTTTA	
AT1G66090-F	TCTGTGGTCACCTCTTTTGCTG	
AT1G66090-R	AACCGGTCCTACGGGTCTTT	
AT1G72940-F	TTCGCCGTCGTTGTTGTCTC	
AT1G72940-R	GATCTTCTTCTCTAGCCTCGTGC	
actin2-F	TTGACAGAGAAGAACAAGGAAGAA	q-PCR (*Arabidopsis* control)
actin2-R	GGAAGAAGATGAGATTGAGGAAGA	
NbActin-F	CGGAATCCACGAGACTACATAC[30]	q_PCR(*Nicotiana* control)
NbActin-R	GGGAAGCCAAGATAGAGC[30]	
18S_F	ACCGTGGCCAGACAAACTAC	q-PCR (*Meloidogyne* control)
18S_R	GATCGCTAGTTGGCATCGTT	

Supplementary Table S5. List of defense response genes that was differentially expressed in the *MiISE5* transgenic plants and the WT plants (FDR ≤ 0.05).

Gene ID	Symbol	Gene annotation	logFC	FDR
AT5G49520	WRKY48	Probable WRKY transcription factor 48	3.19785	0.000000278
AT1G80840	WRKY40	Probable WRKY transcription factor 40	3.334907	2.57E-08
AT3G49530	NAC062	NAC domain-containing protein 62	2.519999	0.0000649
AT4G12720	ATNUDT7	Nudix hydrolase 7	2.135306	0.001108
AT2G39200	MLO12	MLO-like protein	2.12877	0.004353
AT2G33580	LYK5	Protein LYK5	2.368327	0.000288
AT3G20600	NDR1	Protein NDR1	2.568809	0.00015
AT2G30020	AP2C1	PP2C-type phosphatase AP2C1	3.583276	4.11E-09
AT1G17420	LOX3	Lipoxygenase 3, chloroplastic	5.308424	6.41E-17
AT3G44260	CAF1-9	Probable CCR4-associated factor 1 homolog 9	4.056785	2.14E-11
AT1G52030	F-ATMBP	Myrosinase-binding protein 2	-2.09237	0.004186
AT3G25250	OXI1	Serine/threonine-protein kinase OXI1	2.185146	0.004087
AT1G74950	JAZ1	Jasmonate-zim-domain protein 1	1.928403	0.004603
AT5G64900	PEP1	Elicitor peptide 1	2.553458	0.002079
AT5G52020	ERF025	Ethylene-responsive transcription factor ERF025	4.077205	7.66E-09
AT5G66210	CPK28	Calcium-dependent protein kinase 28	2.313317	0.000253
AT2G40140	CZF1	Zinc finger CCCH domain-containing protein 29	2.200878	0.000641
AT5G37770	CML24	Calcium-binding protein CML24	3.592686	2.65E-09
AT1G17380	JAZ5	Jasmonate-zim-domain protein 5	3.908654	8.01E-10
AT2G15890	MEE14	CCG-binding protein 1	-2.06052	0.001987
AT1G20823	ATL80	RING-H2 finger protein ATL80	3.286885	0.000000154
AT5G20230	BCB	blue-copper-binding protein	2.206003	0.000577
AT5G24590	NAC091	NAC domain-containing protein 91	1.97724	0.003648
AT5G13220	JAZ10	Jasmonate-zim-domain protein 10	3.142519	0.000000613

AT3G61190	BAP1	BON1-associated protein 1	4.48735	3.23E-11
AT2G27080	NHL13	NDR1/HIN1-like protein 13	4.154048	1.19E-11
AT3G15210	ERF4	Ethylene-responsive transcription factor 4	1.991583	0.003116
AT1G19180	JAZ5	TIFY10A	2.668486	0.0000105
AT4G31800	WRKY18	WRKY like transcription factor	2.974197	0.000000782
AT4G17490	ERF6	Ethylene-responsive transcription factor 6	3.017079	0.000000924
AT2G34600	JAZ7	Jasmonate-zim-domain protein 7	5.073347	6.45E-14
AT2G35930	PUB23	E3 ubiquitin-protein ligase PUB23	2.713218	0.0000253
AT3G48520	CYP94B3	CYP94B3	5.197636	6.45E-14
AT5G61600	ERF104	Ethylene-responsive transcription factor ERF104	2.09478	0.001673
AT1G29690	CAD1	MACPF domain-containing protein CAD1	1.874435	0.007796
AT5G66070	N/A	RING/U-box superfamily protein	2.097592	0.002378
AT3G22231	PCC1	Cysteine-rich and transmembrane domain-containing protein PCC1	3.233834	0.00000062
AT1G72520	LOX4	Lipoxygenase 4, chloroplastic	3.533435	3.61E-09

Supplementary Table S6. The significantly enriched KEGG terms of DEGs(P ≤0.05).

Items_Details	ID	Number of genes	P-value	Genes
Plant-pathogen interaction	ath04626	10	7.46E-06	WRKY33,WRKY40,WRKY18,WRKY48,CML5, CML38,CML23,CML16,CML24,CPK28
Plant hormone signal transduction	ath04075	6	0.047	JAZ5, JAZ10, XTH22, ARR7,JAZ7,JAZ1
alpha-Linolenic acid metabolism	ath00592	5	4.04E-05	LOX3,LOX4, OPR3, 4CLL5,AOC3
Linoleic acid metabolism	ath00591	2	0.004	LOX3,LOX4
Taurine and hypotaurine metabolism	ath00430	2	0.009	PCO1,PCO2

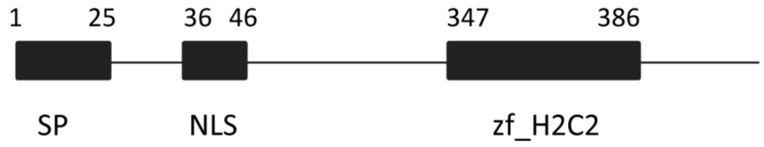

Supplementary Figure 1. Schematic diagram of the protein structure of MiISE5.

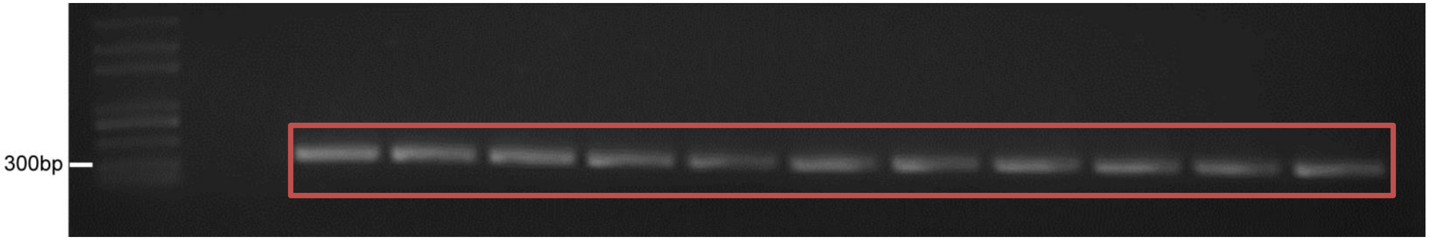

Supplementary Figure 2. Checking of transcripts of coat protein (cp) gene by Reverse transcription PCR.

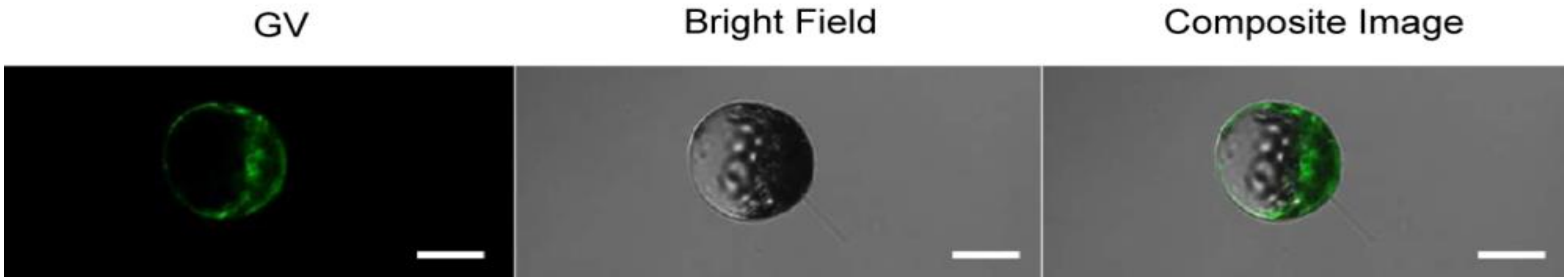

Supplementary Figure 3. Subcellular localization of transiently expressed MiISE5:GFP in Arabidopsis protoplansts. The MiISE5 was localized in the cytoplasm.

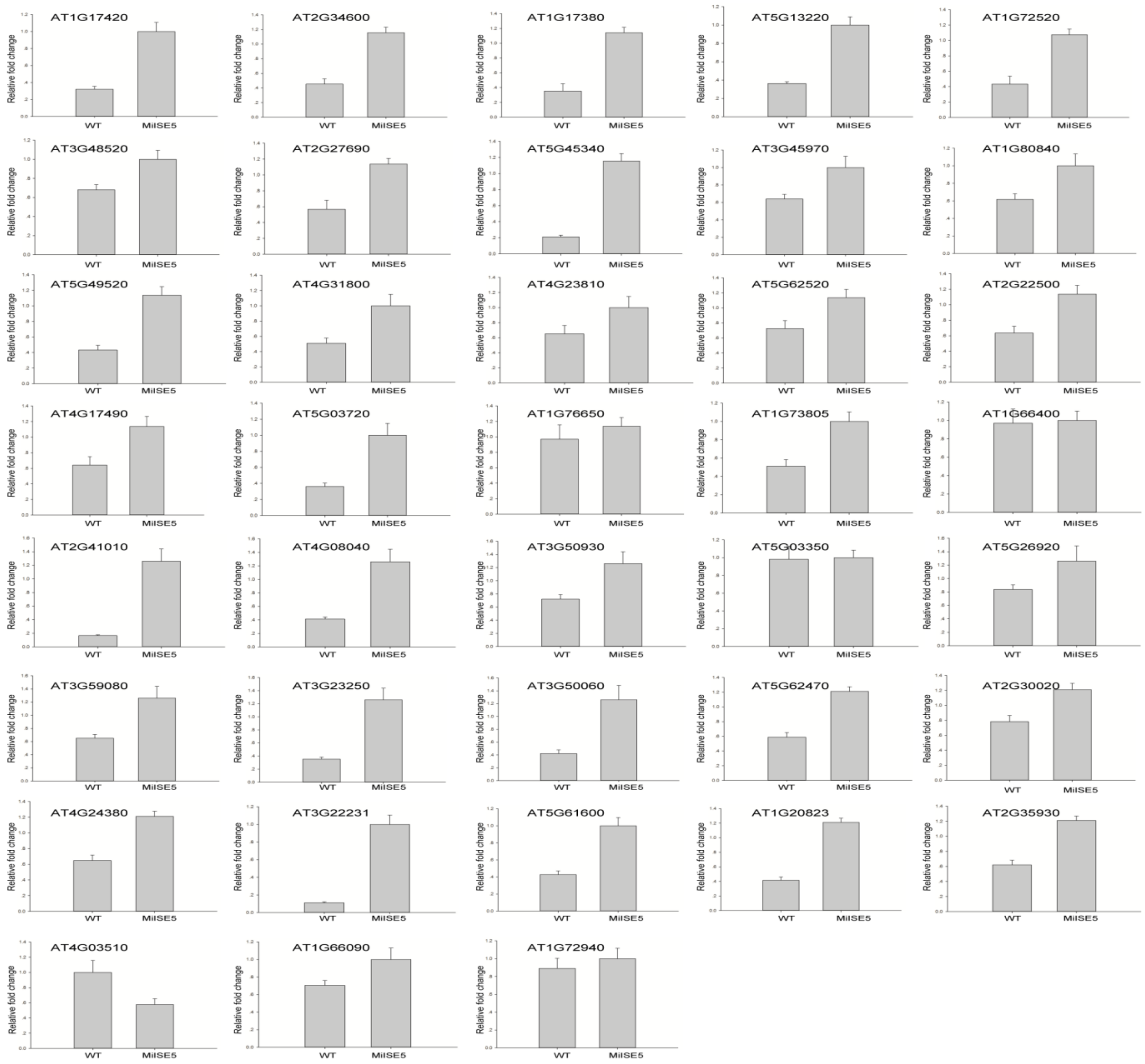
AT1G17420
AT2G34600
AT1G17380
AT5G13220
AT1G72520
AT3G48520
AT2G27690
AT5G45340
AT3G45970
AT1G80840
AT5G49520
AT4G31800
AT4G23810
AT5G62520
AT2G22500
AT4G17490
AT5G03720
AT1G76650
AT1G73805
AT1G66400
AT2G41010
AT4G08040
AT3G50930
AT5G03350
AT5G26920
AT3G59080
AT3G23250
AT3G50060
AT5G62470
AT2G30020
AT4G24380
AT3G22231
AT5G61600
AT1G20823
AT2G35930
AT4G03510
AT1G66090
AT1G72940
Relative fold change
WT
MilSE5

Supplementary Figure 4. Verification of gene expression patterns in WT and *MiISE5* overexpression *Arabidopsis*. 38 DEGs were selected to validate the reliability of the RNA-seq results. The experiments were performed three times with similar results. Actin2 (AT3G18780) was used as internal control gene to normalize the expression of the genes in each corresponding q-PCR sample.

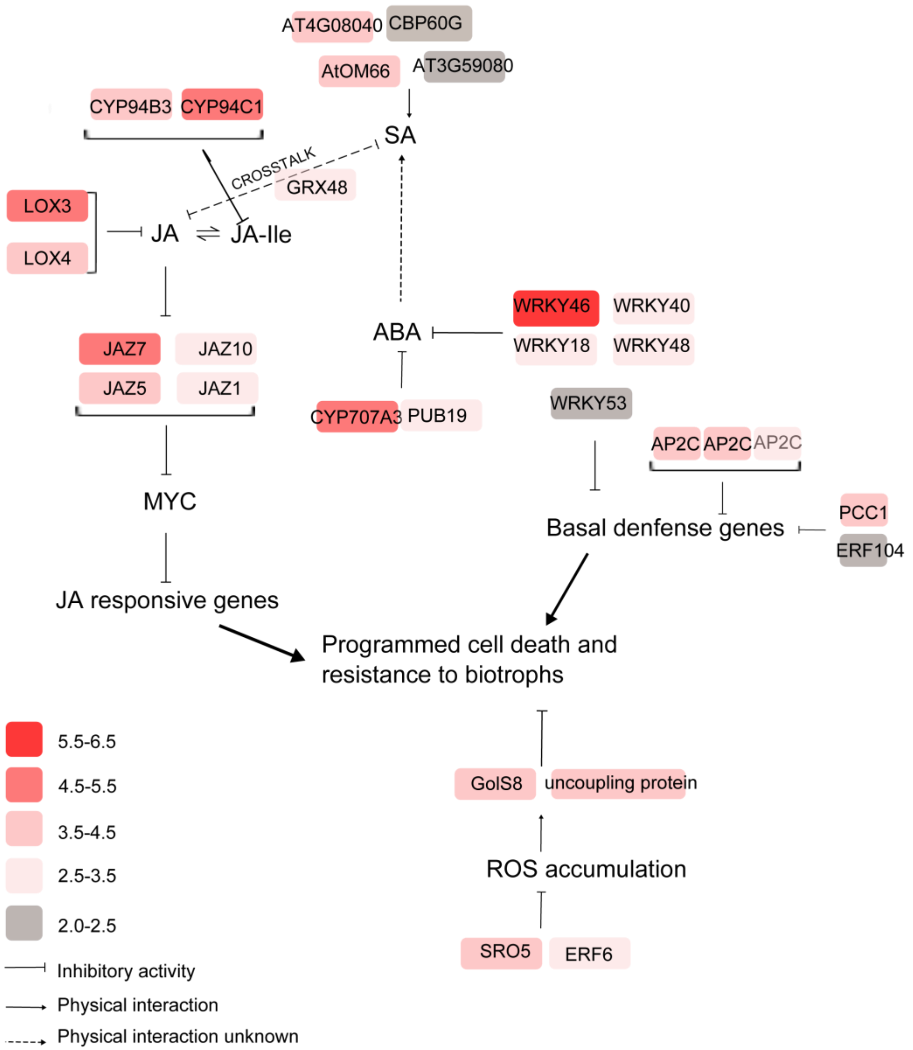

AT4G08040
CBP60G
AtOM66
AT3G59080
CYP94B3
CYP94C1
SA
CROSSTALK
GRX48
LOX3
LOX4
JA
JA-Ile
JAZ7
JAZ10
JAZ5
JAZ1
ABA
WRKY46
WRKY40
WRKY18
WRKY48
CYP707A3
PUB19
WRKY53
AP2C
AP2C
AP2C
MYC
Basal denfense genes
PCC1
ERF104
JA responsive genes
Programmed cell death and resistance to biotrophs
GolS8
uncoupling protein
ROS accumulation
SRO5
ERF6
5.5-6.5
4.5-5.5
3.5-4.5
2.5-3.5
2.0-2.5
Inhibitory activity
Physical interaction
Physical interaction unknown

Supplementary Figure 5. Potential DEGs involved in host defense responses. The color bar represents the expression level (log_2FC) of DEGs.

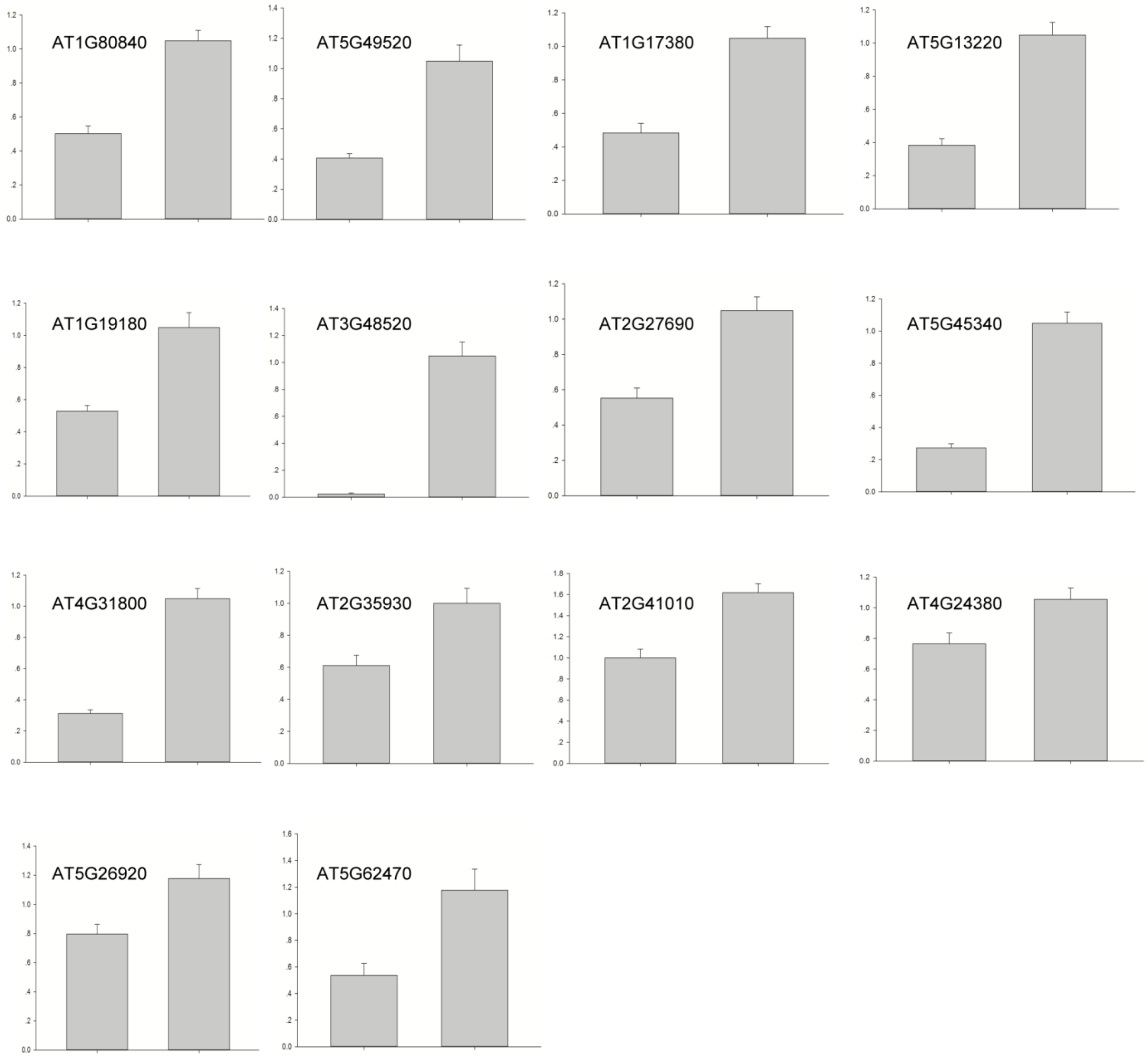
AT1G80840
AT5G49520
AT1G17380
AT5G13220
AT1G19180
AT3G48520
AT2G27690
AT5G45340
AT4G31800
AT2G35930
AT2G41010
AT4G24380
AT5G26920
AT5G62470

Supplementary Figure 6. Verification of gene expression patterns in WT and MiISE5 overexpression Arabidopsis T_5 line. 14 DEGs were selected to validate the reliability of the RNA-seq results. The experiments were performed three times with similar results. Actin2 (AT3G18780) was used as internal control gene to normalize the expression of the genes in each corresponding q-PCR sample.
